# From Mucin to Smart Medicine: Engineering Mucin‐Based Biomaterials for Advanced and Responsive Drug Delivery

**DOI:** 10.1155/adpp/3233459

**Published:** 2026-11-09

**Authors:** Alaa A. Abudayah

**Affiliations:** ^1^ Department of Pharmaceutical Sciences School of Pharmacy Jadara University P.O. Box 733 Irbid 21110 Jordan jadara.edu.jo

**Keywords:** biomaterials, drug delivery, glycoproteins, molecular programming, mucin, mucosomes, nanocarriers, stimuli-responsive delivery

## Abstract

Mucin, a highly glycosylated macromolecular component of mucus, has traditionally been regarded as a biological barrier that restricts drug transport across mucosal surfaces. Growing evidence, however, has repositioned mucin as a functional and engineerable biomaterial with distinctive potential for advanced drug delivery. This review examines the evolution of mucin‐based delivery systems from native mucin and bulk matrices to engineered hydrogels, hybrid materials, micro‐ and nanoparticles, and intrinsically glycosylated nanocarriers such as mucosomes. Particular emphasis is placed on the relationship between mucin molecular architecture and pharmaceutical functionality and on the engineering strategies used to control its material properties, including chemical modification, crosslinking, polymer hybridization, nanoscale assembly, and molecular programming. These approaches have progressively transformed mucin‐based systems from passive drug reservoirs into environmentally responsive and increasingly programmable platforms capable of controlled release, molecular recognition, and on‐demand therapeutic responses. Emerging applications in mucosal and ocular delivery, antimicrobial and biofilm therapy, biologics, and nucleic acid therapeutics further demonstrate the expanding pharmaceutical potential of mucin. Nevertheless, clinical translation remains constrained by source‐dependent heterogeneity, variability in glycosylation and purification, batch‐to‐batch reproducibility, stability, sterilization, scalable manufacturing, and limited in vivo validation. Future progress will require better‐defined mucin materials, standardized characterization and manufacturing, rigorous safety and efficacy assessment, and demonstration of clear therapeutic advantages over established delivery systems. Collectively, advances in mucin engineering, glycoengineering, nanotechnology, and molecular programming may enable the transition of mucin from a biological barrier component to a responsive, programmable, and clinically relevant platform for smart drug delivery.

## 1. Introduction: From Biological Barrier to Functional Drug Delivery Biomaterial

Mucosal surfaces represent some of the most important biological interfaces between the human body and the external environment. Covering the gastrointestinal, respiratory, reproductive, and ocular epithelia, mucus provides a hydrated and dynamic protective layer that simultaneously permits the exchange of essential molecules while restricting the access of potentially harmful particles, microorganisms, and xenobiotics. The functional backbone of this complex biological barrier is formed by mucins, a heterogeneous family of high‐molecular‐weight glycoproteins characterized by extensively O‐glycosylated domains and a chemically diverse molecular architecture [1, 2]. Their dense glycosylation, high water‐binding capacity, ability to establish intermolecular associations, and interactions with biological and synthetic entities enable mucins to generate highly hydrated networks with distinctive barrier, lubricating, viscoelastic, and bioactive properties [1, 2].

From a pharmaceutical perspective, mucus has traditionally been regarded primarily as an obstacle to efficient drug delivery [3]. The mucin‐rich network can interact with therapeutic molecules and particulate carriers through electrostatic, hydrophobic, hydrogen‐bonding, and other noncovalent interactions, thereby influencing their diffusion, retention, and clearance from mucosal surfaces [4]. Consequently, considerable research effort has focused on developing mucoadhesive systems that prolong residence within mucus or, conversely, mucus‐penetrating systems designed to minimize interactions with mucins and facilitate transport toward the underlying epithelium [5, 6]. In this conventional framework, mucin is predominantly considered a physiological barrier that drug delivery technologies must either exploit or overcome [4].

An emerging body of research, however, has begun to invert this paradigm. Rather than treating mucin exclusively as a barrier, investigators have increasingly recognized the glycoprotein itself as a functional and engineerable biomaterial [1, 2]. This conceptual transition is supported by several intrinsic characteristics of mucins that are highly attractive for pharmaceutical material design, including extensive hydration, biological recognition, chemical functionality, biocompatibility, capacity for molecular interactions, and ability to form or participate in supramolecular networks. Purified mucins can therefore serve not only as experimental models of native mucus but also as structural components of hydrogels, matrices, coatings, and particulate drug delivery systems [2, 4, 7]. Importantly, this approach differs fundamentally from conventional mucoadhesive formulations: instead of merely engineering a synthetic carrier to interact with mucin, mucin itself becomes a structural and functional component of the engineered carrier [2, 4].

The development of mucin‐based delivery platforms has consequently progressed from relatively simple matrices toward increasingly sophisticated biomaterial architectures. Chemical modification, covalent and noncovalent crosslinking, polymer hybridization, and nanoscale assembly have enabled mucin to be incorporated into materials with tunable physicochemical and drug‐release properties. Particularly notable is the emergence of mucin‐based nanoparticles. In 2022, Butnarasu et al. introduced mucosomes, intrinsically glycosylated mucin nanoparticles approximately 150–300 nm in size that can accommodate cargos with diverse physicochemical characteristics while retaining functional glycan motifs and intrinsic mucoadhesive properties [4]. This platform illustrates an important shift in formulation philosophy: the biological macromolecule is no longer an inert excipient but contributes directly to the interfacial and biological behavior of the delivery system [8].

Collectively, these advances reflect a broader transition in the role of mucin in drug delivery—from a native component of the biological barrier to a functional biomaterial that can be engineered into increasingly sophisticated delivery platforms. As illustrated in Figure 1, advances in mucin engineering have enabled the progression from bulk matrices and hydrogels to nanoscale carriers, while simultaneously expanding their delivery capabilities from passive and sustained release toward targeted, responsive, and programmable therapeutic systems.

**FIGURE 1:**
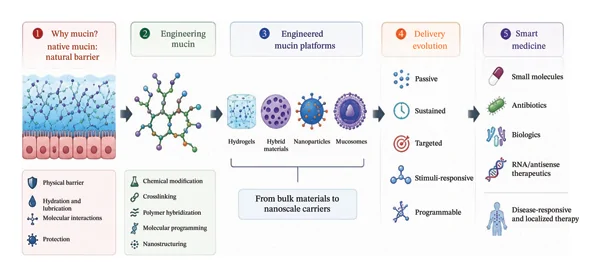
Schematic representation of the conceptual evolution of mucin from a physiological barrier component to an engineered biomaterial for advanced drug delivery. Engineering of mucin through chemical modification, crosslinking, polymer hybridization, molecular programming, and nanostructuring enables diverse carrier architectures and progressively expands functionality from passive release to targeted, stimuli‐responsive, and programmable therapeutic delivery.

More recent developments have moved the field beyond conventional sustained or localized delivery toward responsive and programmable mucin‐based biomaterials. For example, DNA‐mediated crosslinking has been employed to construct mucin hydrogels whose mechanical properties and integrity can be modulated by molecular or thermal triggers. Such systems can undergo controlled gel‐to‐sol transitions and release incorporated particulate cargo on demand, demonstrating how molecular recognition elements can transform mucin networks into trigger‐responsive delivery depots [9]. These advances establish an important bridge between natural biomaterials and the broader concept of “smart” drug delivery, in which the carrier actively responds to defined environmental or molecular signals rather than functioning solely as a passive reservoir.

At the nanoscale, this evolution is becoming even more pronounced. Recent studies have demonstrated that the inherent biological functions of mucin can be preserved and therapeutically exploited after its conversion into nanoparticles. Ciprofloxacin‐loaded mucosomes, for instance, have been investigated against mucus‐associated *Pseudomonas aeruginosa* and *Staphylococcus aureus* infections and biofilms. In this system, the mucin‐based carrier contributed biological activity in addition to serving as a vehicle for the antibiotic, illustrating the possibility of creating delivery platforms in which the carrier and therapeutic cargo exert complementary functions [10]. Most recently, mucin glycoproteins have been engineered into DNA‐stabilized nanoparticles for sequence‐selective antisense therapy against oncogenic microRNAs. In this design, synthetic oligonucleotides simultaneously contribute to nanoparticle stabilization and act as therapeutic recognition elements, enabling sequence‐specific targeting of miRNA‐21 and demonstrating antitumor activity in vivo [11]. Such findings mark a conceptual progression from mucin‐containing formulations to programmable mucin‐based therapeutic systems.

Despite these advances, the development of mucin as a pharmaceutical biomaterial remains at an early stage compared with established natural polymers such as alginate, chitosan, hyaluronic acid, and collagen. Important challenges remain regarding mucin source and purification, molecular heterogeneity, preservation of glycosylation and biological functionality, batch‐to‐batch reproducibility, material characterization, sterilization, scalable manufacturing, and regulatory standardization [2, 12]. These issues become increasingly relevant as mucin‐based technologies evolve from experimental hydrogels and nanoparticles toward more complex responsive and biologically active therapeutic systems.

Against this background, this critical narrative review examines the evolution of mucin from a biological barrier component to an engineered platform for advanced and responsive drug delivery. Emphasis is placed on mucin structure–function relationships, engineering strategies, carrier architectures, cargo loading and release, smart and programmable delivery, therapeutic applications, and key manufacturing, safety, and translational challenges. To provide a transparent evidence base, PubMed/MEDLINE, Scopus, and Web of Science were searched for peer‐reviewed English‐language studies published between January 2000 and September 2026 using combinations of terms related to mucin biomaterials, hydrogels, nanoparticles, mucosomes, drug delivery, responsiveness, molecular programming, glycans, and mucoadhesion. Reference lists of relevant primary studies and reviews were also screened. Priority was given to studies in which mucin constituted a structural or functional component of the delivery system, whereas studies using mucin solely as a diffusion barrier or mucoadhesion‐testing substrate were excluded. As this is a critical narrative rather than systematic review, studies were selected according to relevance to the review objectives, with particular consideration of mucin source, formulation architecture, therapeutic cargo, response mechanism, experimental model, and level of biological evidence. Ultimately, the review seeks to define when and how mucin provides meaningful functionality in drug delivery and to identify the evidence required to advance mucin‐based materials toward smart medicine.

## 2. Mucin as a Pharmaceutical Building Block: Structure, Chemistry, and Functional Attributes

The suitability of mucin as a pharmaceutical biomaterial originates from its distinctive molecular architecture, in which a protein backbone, densely glycosylated domains, charged residues, hydrophobic regions, and reactive functional groups coexist within the same macromolecule. Unlike many conventional pharmaceutical polymers whose functionality is dominated by a relatively limited number of repeating chemical groups, mucins exhibit pronounced molecular heterogeneity and multifunctionality. This structural complexity governs hydration, molecular association, viscoelasticity, interfacial adhesion, molecular recognition, and interactions with therapeutic compounds and biological surfaces [1, 2, 13]. From a drug delivery perspective, these characteristics provide a versatile molecular basis for constructing hydrated matrices, crosslinked networks, hybrid materials, and particulate carriers [1, 2, 13]. Understanding how mucin structure translates into pharmaceutical functionality is therefore essential for the rational engineering of mucin‐based delivery systems.

### 2.1. Molecular Architecture and Glycosylation

Mucins are high‐molecular‐weight glycoproteins characterized by a protein backbone containing domains rich in proline, threonine, and serine (PTS domains). Serine and threonine residues are extensively modified with O‐linked oligosaccharides, producing the characteristic extended, bottle brush–like architecture of mucin molecules [1, 2, 13]. The carbohydrate‐rich domains constitute a substantial fraction of mucin molecular mass and commonly contain N‐acetylgalactosamine (GalNAc), N‐acetylglucosamine (GlcNAc), galactose (Gal), and fucose (Fuc), together with terminal sialic‐acid residues and sulfate groups [1, 2, 13]. Dense glycosylation not only determines molecular conformation but also promotes hydration, protects the protein backbone, and contributes to the chemical and biological diversity of mucins [1, 2, 13]. The principal structural features of mucin, including its extensively O‐glycosylated PTS domain and cysteine‐rich regions involved in intermolecular assembly [14], are illustrated in Figure 2.

**FIGURE 2:**
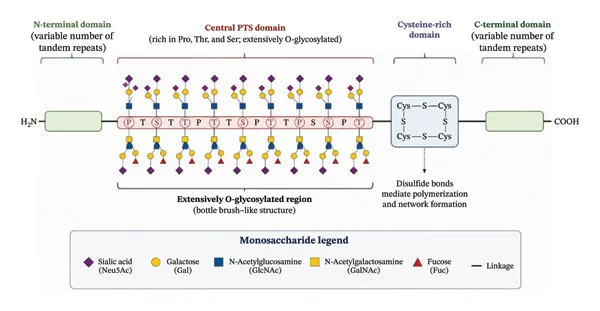
Molecular architecture and principal structural features of gel‐forming mucin. The mucin macromolecule comprises a densely O‐glycosylated proline‐, threonine‐, and serine‐rich (PTS) domain that forms the characteristic bottle brush architecture, while cysteine‐rich regions mediate disulfide‐linked polymerization and network formation. The heterogeneous glycan composition governs hydration, charge‐dependent interactions, molecular recognition, and protection of the protein backbone.

This highly glycosylated architecture is directly responsible for several properties relevant to pharmaceutical formulation. Dense hydrophilic glycans promote extensive association with water, whereas terminal sialic‐acid and sulfate groups confer negative charge and provide sites for electrostatic interactions [1, 2, 13]. These characteristics are also sensitive to the surrounding physicochemical environment. Recent molecular simulations demonstrated that glycosylation and interactions with cations, particularly Na^+^ and Ca^2+^, can modulate the dimensions and conformational behavior of mucin domains [15]. Such observations provide a molecular basis for the influence of ionic conditions on mucin organization and material behavior.

In contrast to the densely glycosylated PTS regions, less‐glycosylated protein regions provide comparatively hydrophobic domains, while cysteine‐rich regions in gel‐forming mucins participate in intermolecular disulfide bonding and higher‐order assembly [2, 13, 16]. Thus, an individual mucin macromolecule combines hydrophilic, hydrophobic, charged, and chemically reactive regions, providing multiple opportunities for drug association, polymer interaction, crosslinking, molecular recognition, and material engineering.

Importantly, mucin architecture is not uniform across all mucins. Mucin subtype, anatomical origin, species, glycosylation profile, and purification procedure can alter molecular composition and physicochemical behavior [13, 17]. This is particularly relevant in pharmaceutical research, where purified porcine gastric mucin (PGM) and bovine submaxillary mucin (BSM) are frequently used as experimental materials [18]. A direct comparison published in 2024 demonstrated compositional differences between these mucins and showed that their interactions with pharmaceutical polymers were not equivalent [17]. Mucin source should therefore be regarded as an important material attribute rather than a trivial experimental variable.

### 2.2. Physicochemical and Rheological Properties

The molecular architecture of mucin gives rise to a distinctive combination of hydration, viscoelasticity, surface activity, and environmental responsiveness. Among these properties, hydration is fundamental. Extensive glycosylation enables mucin molecules to associate with substantial quantities of water and maintain highly hydrated molecular environments [1, 2, 13]. Upon intermolecular association or engineered crosslinking, mucins can form three‐dimensional hydrated networks, providing a physicochemical basis for their use in hydrogels and other water‐rich drug delivery matrices [1, 2].

Mucin‐containing networks exhibit both viscous and elastic characteristics, and their rheological properties depend on mucin concentration, molecular organization, crosslink density, pH, ionic composition, and applied mechanical stress [16, 19]. Native mucus therefore behaves as a complex viscoelastic material rather than a simple viscous liquid. Its rheological behavior may include shear‐dependent flow, elastic responses, and yield behavior, reflecting the organization of the mucin network together with contributions from proteins, lipids, nucleic acids, ions, cells, and other constituents [16, 19]. These characteristics influence molecular and particulate transport through mucus and are consequently relevant to drug delivery performance [20].

For engineered mucin materials, rheological properties have direct implications for drug retention and release. Network density and crosslinking regulate molecular mobility and effective mesh dimensions, thereby influencing the diffusion of incorporated therapeutic compounds [16]. Increased network connectivity may improve mechanical stability and drug retention while slowing diffusion, whereas more hydrated and loosely crosslinked networks generally permit greater molecular mobility. Rheological properties can therefore become formulation parameters through which structural persistence, residence time, and release kinetics are adjusted.

Environmental conditions provide an additional level of control over mucin behavior. Variations in pH and ionic composition can modify electrostatic screening, mucin conformation, intermolecular association, and network organization [21]. Such sensitivity is particularly relevant to mucosal delivery routes characterized by dynamic physicochemical conditions, including the gastrointestinal tract. Consequently, the intrinsic environmental responsiveness of mucin provides a physicochemical foundation that can subsequently be amplified through chemical modification and crosslinking to generate engineered stimuli‐responsive systems [22].

An important distinction must nevertheless be maintained between native mucus and purified mucin‐based materials. Native mucus is a multicomponent biological secretion, whereas purified mucin preparations may undergo structural or compositional changes during isolation and processing [17]. Rheological observations obtained from physiological mucus therefore cannot always be directly extrapolated to purified mucin formulations, emphasizing the need for material‐specific physicochemical characterization.

### 2.3. Mucoadhesion and Biological Interactions

Mucoadhesion arises from a combination of molecular and physical interactions rather than a single binding mechanism. Electrostatic attraction, hydrogen bonding, hydrophobic interactions, van der Waals forces, polymer‐chain interpenetration, and, in appropriately functionalized materials, covalent interactions may all contribute to association with mucin [21, 23]. The relative contribution of each mechanism depends on polymer chemistry, molecular weight, charge density, mucin composition, hydration, pH, and ionic conditions [24].

These interactions are pharmaceutically important because association with the mucus layer can increase the residence time of a formulation at a mucosal surface, thereby prolonging localized drug exposure or increasing the time available for absorption [21, 23, 25]. Chitosan provides a representative example: under suitable pH conditions, its protonated amino groups can interact with negatively charged mucin components through electrostatic attraction, supplemented by hydrogen bonding and other noncovalent interactions [17, 21].

Importantly, mucoadhesion should not be regarded solely as a property of the pharmaceutical formulation. The molecular characteristics of the mucin substrate itself can substantially influence the apparent strength of interaction. In a direct comparison of PGM and BSM, polyethylene oxide showed relatively weak interactions with both mucins, whereas chitosan interacted with both but exhibited stronger interaction with BSM [17]. These differences demonstrate that the mucin model selected for pharmaceutical testing can influence conclusions regarding mucoadhesive performance [26].

Mucin‐based carriers introduce a conceptually different approach from conventional mucoadhesive systems. In conventional systems, mucin represents the biological substrate with which an administered polymer or particle interacts. In mucin‐based carriers, however, mucin itself becomes a structural and interfacial component of the delivery platform. Intrinsic features such as hydration, surface charge, glycan‐mediated interactions, and molecular recognition can therefore become functional properties of the engineered carrier [1, 2].

Mucin glycans are particularly important in this context because they participate in biological recognition and host–microorganism interactions. Specific glycan motifs can interact with lectins, microbial adhesins, receptors, and other biological molecules, demonstrating that mucin surfaces possess biological information in addition to physicochemical functionality [27]. Preservation of relevant glycan structures during purification and carrier fabrication may therefore enable engineered mucin systems to retain biological interactions that are difficult to reproduce using conventional synthetic polymers.

### 2.4. Drug–Mucin Interactions

The chemical heterogeneity of mucin creates multiple potential interaction environments for therapeutic molecules. Depending on drug chemistry, drug–mucin interactions may involve electrostatic attraction or repulsion, hydrogen bonding, hydrophobic association, steric effects, or combinations of these mechanisms [28]. Mucin should therefore not be regarded as an inert polymeric scaffold through which incorporated drugs simply diffuse. Instead, molecular affinity between mucin and therapeutic cargo can influence drug partitioning, retention, mobility, and release.

Recent experimental evidence provides strong support for this concept. A systematic investigation of interactions between therapeutic compounds and the human polymeric mucins MUC2, MUC5AC, and MUC5B demonstrated substantial drug‐ and mucin‐dependent differences in binding and aggregation behavior [28]. Paclitaxel, rifampicin, and theophylline promoted aggregation of selected polymeric mucins under the investigated conditions, while binding behavior differed among individual mucin subtypes [28]. Furthermore, alterations in cellular mucin expression affected the cytotoxic activity of selected drugs, including paclitaxel and cyclosporin A, indicating that drug–mucin interactions may have pharmacological consequences extending beyond simple physical retention [28].

For engineered mucin‐based delivery systems, these interactions present both an opportunity and a potential limitation. Strong but reversible association may enhance cargo loading, reduce premature drug loss, and contribute to sustained release. Conversely, excessively strong binding may restrict release and reduce the therapeutically available drug fraction. Hydrophobic regions of the protein backbone may contribute to interactions with poorly water‐soluble compounds, whereas charged glycan and protein domains provide potential interaction sites for ionic molecules and biomacromolecules [1, 2, 13, 28]. The balance between affinity and reversibility is therefore an important determinant of the performance of mucin‐based formulations.

Drug–mucin interactions should consequently be considered during carrier design in much the same way that polymer–drug compatibility is evaluated in conventional polymeric delivery systems. Relevant drug characteristics include molecular size, charge, hydrophobicity, hydrogen‐bonding capacity, and conformational stability, while mucin subtype, glycosylation, charge, and processing history may determine the complementary binding environment [28]. This consideration becomes increasingly important as mucin‐based platforms expand beyond conventional small molecules toward antibiotics, proteins, antibodies, and nucleic acid therapeutics.

### 2.5. Structure–Property–Delivery Function Relationships

Taken together, the pharmaceutical potential of mucin can be understood through a direct structure–property–delivery function relationship. Dense O‐glycosylation promotes hydration and molecular extension; terminal sialylated and sulfated glycans contribute negative charge, ionic sensitivity, and molecular interactions; less‐glycosylated protein regions provide opportunities for hydrophobic association; and cysteine‐rich regions support intermolecular assembly and network formation [13, 29]. These molecular characteristics collectively determine material‐level properties including hydration, viscoelasticity, adhesion, molecular permeability, drug‐binding capacity, and environmental responsiveness.

The importance of this continuum lies in the fact that modification of a single structural characteristic may simultaneously alter several delivery‐relevant properties. Increasing crosslink density, for example, can strengthen a mucin network and improve structural persistence while restricting molecular diffusion and prolonging drug release [1, 13]. Modification of charged groups can alter drug affinity, polymer interactions, and interfacial behavior, whereas preservation or alteration of glycosylation may affect hydration, molecular conformation, surface charge, and biological recognition [1, 2].

The same principle applies when mucin is engineered at the nanoscale. Assembly of mucin molecules into particulate systems translates intrinsic molecular characteristics into particle‐level properties, including surface chemistry, charge, colloidal behavior, cargo association, and biological interactions [1, 13]. Nanoengineering therefore does not replace the inherent functionality of mucin; rather, it reorganizes and exploits these molecular features at a different structural scale [30].

Crucially, these relationships explain why mucin cannot be treated as a single standardized pharmaceutical polymer. Mucin subtype, anatomical and species origin, glycosylation profile, molecular integrity, purification procedure, and processing history can influence the properties of the resulting material [17]. The different polymer‐binding behaviors observed for PGM and BSM, together with the mucin subtype–dependent drug interactions demonstrated for MUC2, MUC5AC, and MUC5B, provide experimental evidence for this principle [28].

These structure–property relationships establish the mechanistic foundation for the engineering strategies discussed in the following section. Chemical functionalization, covalent and physical crosslinking, polymer hybridization, molecular programming, and nanoengineering can all be viewed as approaches for deliberately manipulating mucin structure and intermolecular interactions to generate specific pharmaceutical functions. Accordingly, the transition from native mucin to an advanced drug delivery platform depends not simply on incorporating mucin into a formulation, but on understanding, preserving, and deliberately engineering the relationship between mucin structure and delivery performance.

To place mucin in the context of established delivery materials, Table 1 provides a qualitative comparison across attributes that are particularly relevant to pharmaceutical development. The comparison highlights a central trade‐off: mucin offers unusually rich glycan‐mediated biological functionality and heterogeneous drug‐interaction sites, but currently has weaker standardization and manufacturing maturity than widely used polymers and lipid‐based carriers. These comparisons are context dependent and should be interpreted as design considerations rather than universal performance rankings.

**TABLE 1:** Qualitative comparison of mucin‐based drug delivery materials with established natural, synthetic, and lipid‐based platforms.

Material/platform	Loading and release control	Biodegradability/biocompatibility	Reproducibility and stability	Immunological/source considerations	Translational position	References
Mucin‐based systems	Broad interaction chemistry; tunable by crosslinking and nanoengineering	Generally favorable in reported preclinical studies; proteolytically degradable	Source‐, glycosylation‐, purification‐, and batch‐dependent; storage/sterilization needs standardization	Animal‐derived contaminants and xenogeneic glycans require control; intrinsic bioactivity may be advantageous or confounding	Emerging; limited standardized manufacturing and in vivo validation	[1, 2, 31]
Alginate	Good for ionically crosslinked depots; loading depends on cargo chemistry	Widely used natural polysaccharide; degradation depends on formulation and modification	Commercially accessible; molecular‐weight/composition variability remains	Generally low immunogenicity when purified; impurity control remains relevant	Relatively mature for hydrogel/encapsulation applications	[32, 33]
Chitosan	Strong mucoadhesion and ionic complexation; pH‐dependent solubility	Biodegradable; extensive mucosal‐delivery experience	Degree of deacetylation and molecular weight influence performance	Source and purity can affect biological response	Well studied preclinically; formulation and solubility constraints remain	[32, 34]
Hyaluronic acid	Highly hydrated matrices; readily functionalized for controlled release	Endogenous and biodegradable	Defined grades available, although molecular weight strongly affects behavior	Generally favorable biocompatibility; modification chemistry must be controlled	Broad biomedical use and comparatively strong translational familiarity	[35]
Collagen/gelatin	Useful matrices and hydrogels; loading often diffusion/crosslink dependent	Biodegradable and cell‐interactive	Animal‐source and batch variability; mechanical stability often requires crosslinking	Potential xenogeneic and immunological concerns depend on source/purification	Extensive biomaterial use; source control is important	[36]
PEG‐based systems	Highly tunable network and surface chemistry; limited intrinsic cargo affinity unless functionalized	Biocompatible but generally not inherently biodegradable unless designed with labile linkages	High chemical reproducibility and scalable synthesis	Low intrinsic bioactivity; repeated exposure and anti‐PEG responses can be relevant in some contexts	Highly established synthetic platform	[37]
Lipid‐based carriers	High versatility for small molecules and nucleic acids; release depends on lipid composition	Biocompatible lipids available; degradation pathways well characterized for many systems	Manufacturing, oxidation, leakage, and storage stability require control but processes are well developed	Composition‐dependent innate immune effects possible	High translational maturity, including approved products	[38]

*Note:* Performance is formulation‐ and application‐dependent; the table summarizes general design and translational considerations rather than assigning superiority.

## 3. Engineering Mucin‐Based Drug Delivery Platforms: From Bulk Biomaterials to Programmable Nanocarriers

The transformation of mucin from a naturally occurring glycoprotein into a pharmaceutical delivery material begins with the selection and preparation of the biological starting material. Unlike conventional synthetic polymers with relatively well‐defined compositions, mucin is structurally heterogeneous, and its properties depend on mucin subtype, anatomical and species origin, glycosylation profile, isolation procedure, purification conditions, and subsequent processing [1, 17]. These variables can influence molecular integrity, charge distribution, glycan presentation, intermolecular interactions, and rheological behavior and may ultimately affect the performance of the resulting delivery system. Therefore, mucin source and quality should be regarded as integral components of material design rather than merely experimental details.

Mucins employed in biomaterial and pharmaceutical research have been obtained from different biological sources, with commercially available PGM and BSM among the most frequently investigated preparations [1, 17]. However, commercial mucins should not automatically be regarded as chemically or functionally interchangeable. Isolation and purification can modify macromolecular organization and associated components, while source‐dependent variations in composition can substantially affect interactions with pharmaceutical polymers [17]. Characterization of the starting material—including source, purity, molecular integrity, glycosylation, charge, and relevant physicochemical properties—is therefore important for reproducibility and rational formulation design.

Native mucin already possesses several attributes advantageous for drug delivery, including high hydration, extensive glycosylation, heterogeneous charged and hydrophobic domains, and multiple sites for molecular interaction. Nevertheless, native mucin alone may not provide the mechanical stability, structural persistence, reproducible assembly, or precisely controlled responsiveness required for advanced pharmaceutical applications [1, 2]. Engineering approaches have therefore progressively transformed mucin from a biological macromolecule into a structurally controllable delivery material.

Importantly, this evolution has occurred along two interconnected dimensions: material engineering and carrier architecture. Chemical functionalization and crosslinking initially enabled stabilization of mucin into bulk networks and hydrogels; hybridization and reversible molecular interactions subsequently introduced greater mechanical and environmental control; molecular programming added sequence‐specific responsiveness; and nanoscale engineering ultimately reorganized mucin into particulate carriers in which its glycans and molecular recognition features become properties of the carrier interface [7, 9, 31, 39–43]. The overall evolution of these systems—from native mucin and bulk matrices to engineered hydrogels, responsive hybrid systems, molecularly programmed biomaterials, and intrinsically functional nanocarriers—is illustrated in Figure 3. The principal engineering strategies underlying this progression, together with their mechanisms, advantages, limitations, and resulting delivery platforms, are comparatively summarized in Table 2. Collectively, these developments demonstrate that the evolution of mucin‐based delivery systems should be understood not simply as a reduction in carrier size, but as a progressive integration of material architecture, molecular engineering, responsiveness, and biological functionality.

**FIGURE 3:**
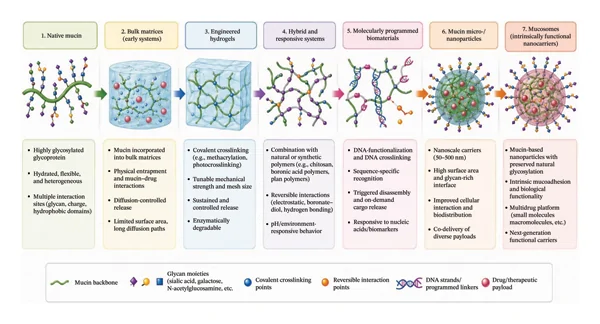
Engineering strategies for transforming native mucin into functional drug delivery biomaterials. Chemical and physical crosslinking, polymer hybridization, glycan‐ and DNA‐mediated interactions, and nanoengineering progressively enable the development of mucin‐based hydrogels, responsive systems, micro‐/nanoparticles, and mucosomes with controlled structural, interfacial, and delivery properties.

**TABLE 2:** Major strategies for engineering native mucin into functional and programmable drug delivery biomaterials.

Engineering approach	Engineering principle	Key advantages	Limitations/engineering considerations	Resulting functionality or platform	Representative studies
Chemical functionalization	Modification of available mucin functional groups to introduce reactive or polymerizable moieties	Expands mucin chemistry; enables conjugation and subsequent crosslinking	Excessive modification may alter charge, glycan accessibility, or native functionality	Functionalized mucin precursors for further engineering	[7]
Covalent crosslinking	Formation of permanent intermolecular bonds between mucin chains	High structural stability; tunable network density and permeability	Irreversible network; crosslinker residues and biocompatibility must be considered	Stable mucin hydrogels and networks	[7]
Methacrylation and photocrosslinking	Introduction of methacryloyl groups followed by light‐induced polymerization	Temporal control over crosslinking; adaptable to bulk and nanoscale systems	Requires compatible photoinitiator and irradiation; potential alteration of native functionality	Photocrosslinked hydrogels and mucin nanoparticles	[7, 40]
Physical/electrostatic assembly	Reversible hydrogen bonding, electrostatic, hydrophobic, ionic, and entanglement interactions	Mild processing; reversible and potentially environmentally responsive	Greater sensitivity to pH, ionic strength, and dilution	Dynamic networks and mucus‐mimicking materials	[31]
Glycan‐mediated dynamic crosslinking	Reversible interactions involving cis‐diol‐containing mucin glycans	Exploits native glycosylation; self‐healing and responsive behavior	Stability depends on environmental conditions and interaction chemistry	Adaptive and reversible mucin hydrogels	[42, 43]
Polymer–mucin hybridization	Combination of mucin with complementary natural or synthetic polymers	Integrates mucin biofunctionality with improved mechanics or responsiveness	Greater compositional, manufacturing, and characterization complexity	Hybrid hydrogels and microparticles	[41, 42]
DNA‐mediated molecular programming	Sequence‐specific nucleic acid interactions function as crosslinks and/or molecular triggers	Molecular recognition; programmable disassembly and triggered release	Nuclease susceptibility, cost, stability, and manufacturing complexity	Responsive hydrogels and programmed nanocarriers	[9, 11]
Self‐assembly and nanoparticle engineering	Nanoscale organization and stabilization of mucin molecules	High interfacial area; cargo loading; glycosylated surface; potential cellular interaction	Colloidal stability, reproducibility, sterilization, and scale‐up remain important	Mucin nanoparticles, programmed nanocarriers, and mucosomes	[4]

### 3.1. Mucin‐Based Matrices and Early Bulk Delivery Systems

The earliest and conceptually simplest use of mucin as a pharmaceutical material involves its incorporation into bulk matrices capable of retaining therapeutic molecules and regulating their release. In such systems, mucin contributes both structural and molecular functions. Its extensively glycosylated domains support hydration and water uptake, whereas charged and comparatively hydrophobic regions provide chemically heterogeneous sites capable of interacting with different classes of therapeutic compounds [1, 2].

Drug retention within these matrices may consequently result from the combined influence of mucin–drug interactions, molecular entanglement, matrix hydration, and diffusion through the polymer‐rich environment. This represents an important distinction from studies in which mucin is used merely as a model substrate to assess mucoadhesion: in a mucin‐based matrix, mucin itself forms part of the delivery material.

The matrix concept has subsequently been expanded through copolymerization and crosslinking. Aslam et al. developed stimuli‐responsive quince/mucin co‐poly(methacrylate) hydrogel matrices for controlled delivery of acyclovir sodium [44]. The system exhibited pH‐dependent swelling and controlled drug‐release behavior, illustrating how incorporation of mucin into an engineered polymeric matrix can convert a relatively simple drug reservoir into an environmentally responsive delivery system.

Bulk matrices nevertheless have inherent architectural limitations. Their relatively low surface‐area‐to‐volume ratio and long diffusional pathways mean that drug delivery is governed predominantly by swelling, diffusion, matrix relaxation, and degradation. These characteristics can be advantageous for localized and prolonged release but provide comparatively limited control over cellular interactions or nanoscale transport. The need for more precisely tunable three‐dimensional networks therefore stimulated the development of engineered mucin hydrogels.

### 3.2. Engineering Mucin Hydrogels: From Covalent Stabilization to Dynamic Networks

Hydrogels represent one of the most established architectures for converting mucin into a functional delivery biomaterial. Their highly hydrated three‐dimensional structure is particularly compatible with the natural physicochemical characteristics of mucin, while crosslinking provides a means of controlling network stability, mechanical properties, mesh size, degradation, and cargo diffusion.

A landmark study by Duffy et al. demonstrated that mucin could be functionalized with methacrylic groups and subsequently crosslinked by ultraviolet irradiation in the presence of a free‐radical photoinitiator to generate robust mucin hydrogels [7]. The resulting materials exhibited an elastic modulus comparable to that of soft mammalian tissues and were susceptible to proteolytic degradation. Importantly, the hydrogels enabled sustained delivery of both hydrophobic paclitaxel and hydrophilic polymyxin B, demonstrating that mucin’s chemically heterogeneous structure could support therapeutics with substantially different physicochemical characteristics [7].

This work established chemical functionalization and covalent crosslinking as important tools for converting native mucin into structurally persistent drug reservoirs. However, permanent covalent networks provide only one level of control. Subsequent approaches have incorporated physical and reversible interactions to generate materials capable of dynamic rearrangement or environmental responsiveness. Tandem chemical and physical crosslinking, for example, has been employed to produce mucus‐mimicking mucin hydrogels with adjustable rheological characteristics [31]. In this system, chemical crosslinking was combined with freeze–thaw–induced physical crosslinking to improve the structural properties of mucin hydrogels.

Particularly interesting is the emerging use of mucin glycans as active engineering elements. Rather than regarding glycosylation solely as a biological characteristic that should be preserved during processing, carbohydrate functionalities can be exploited as reversible interaction sites. Nakahata et al. demonstrated the formation of mucin‐containing hybrid networks through interactions with a boronic acid–bearing polymer [42]. More recently, Nakamura et al. exploited reversible borate–diol interactions involving native mucin glycans to generate dynamic mucin hydrogels with reversible, self‐healing, and adhesive characteristics [43].

The evolution of mucin hydrogels therefore illustrates a broader transition from native mucin to stabilized networks and, subsequently, to dynamic and responsive biomaterials. This progression is significant because the hydrogel gradually changes from being a passive depot through which drug molecules diffuse into a material whose molecular architecture can participate directly in determining material behavior and, potentially, therapeutic release.

### 3.3. Hybrid and Composite Mucin Systems

Although mucin provides several biologically valuable properties, it may not independently provide the mechanical strength, processability, environmental stability, or stimulus responsiveness required for every pharmaceutical application. Hybridization addresses this limitation by combining mucin with natural or synthetic polymers that contribute complementary physicochemical functions [45].

A particularly rational strategy is to engineer a second polymer to interact specifically with mucin’s existing molecular features. Nakahata et al., for example, developed a bio‐synthetic hybrid hydrogel composed of mucin and a synthetic polymer carrying boronic acid functionalities [42]. Reversible interactions between the polymer and carbohydrate functionalities of mucin enabled network formation under physiological conditions. Such systems illustrate an important design principle: rather than extensively modifying mucin itself, a complementary polymer can be engineered to recognize and organize native mucin into a functional network.

Natural polymer combinations provide another approach. Chitosan is particularly attractive because its cationic groups can participate in interactions with negatively charged mucin domains. Malatani et al. exploited chitosan and mucin to produce pH‐responsive hydrogel microparticles for controlled delivery of tofacitinib [41]. The resulting system combined polymer hybridization with particulate architecture and pH‐responsive drug delivery.

Plant‐derived polymers have likewise been combined with mucin to generate responsive delivery matrices. Quince/mucin co‐poly(methacrylate) hydrogels were investigated for controlled delivery of acyclovir sodium [44], while *Delonix regia*/mucin co‐poly(acrylate) hydrogels were subsequently developed for sustained delivery of metformin HCl [46]. The latter displayed pH‐responsive swelling and sustained drug release, illustrating the potential of mucin‐containing hybrid networks for oral delivery.

Hybridization therefore expands the functional design space of mucin‐based delivery. Ideally, each component should have a clearly defined role: mucin contributes hydration, glycosylation, molecular interactions, and biological functionality, whereas the complementary polymer supplies a specific property that mucin alone cannot adequately provide.

This advantage must, however, be balanced against increased formulation complexity. Polymer composition, molecular weight, component ratio, charge density, crosslinking conditions, and batch variability can all influence the final material. Consequently, hybridization should provide a demonstrable functional benefit rather than merely increasing compositional complexity.

### 3.4. Molecularly Programmed Mucin Biomaterials

A major conceptual advance in mucin engineering has been the introduction of molecular components that function not only as structural elements but also as information‐responsive triggers. DNA is particularly attractive in this context because complementary base pairing allows highly specific molecular recognition to be incorporated into material architecture.

Kimna et al. developed mucin‐based nanocarriers in which DNA strands were incorporated into the carrier design to enable sequence‐dependent intracellular drug release [11]. Therapeutic release could be initiated in the presence of a specific trigger sequence, and the concept was extended to an endogenous microRNA sequence associated with disease. Thus, molecular recognition was translated directly into intracellular cargo release, providing an important demonstration of autonomous, biomarker‐responsive mucin‐based delivery.

This concept was subsequently extended to macroscopic mucin networks. Henkel et al. developed DNA‐crosslinked mucin hydrogels in which nucleic acid strands functioned as reversible structural elements [9]. Addition of complementary displacement strands induced controlled network disintegration and the release of incorporated particles.

These systems illustrate a fundamental evolution in the role of crosslinking:

 Structural crosslinking ⟶ molecular recognition ⟶ selective disassembly ⟶ triggered cargo release.

DNA‐mediated engineering is therefore qualitatively different from simply increasing the mechanical stability of a mucin carrier. The structural component can simultaneously function as a molecular recognition element capable of converting a defined biological signal into a material response. This represents one of the clearest bridges between conventional mucin biomaterials and the concept of programmable or “smart” drug delivery.

Nevertheless, nucleic acid–based programming introduces additional translational challenges, including oligonucleotide stability, susceptibility to nuclease degradation, manufacturing complexity, cost, and regulatory considerations. These factors will require careful evaluation if molecularly programmed mucin systems are to progress beyond proof‐of‐concept applications. In addition, sequence‐programmed systems require stringent scrambled‐sequence and nonresponsive controls, demonstration of trigger specificity in protein‐ and nuclease‐rich biological fluids, assessment of off‐target hybridization, intracellular trafficking, persistence of the oligonucleotide component, biodistribution, and repeat‐dose safety. Current demonstrations should therefore be interpreted as proof‐of‐concept evidence rather than as established clinical readiness.

### 3.5. Mucin‐Based Micro‐ and Nanoparticles: Translating Mucin Functionality to the Nanoscale

The transition from bulk networks to particulate systems represents an important architectural shift in mucin‐based delivery. In a macroscopic matrix or hydrogel, mucin predominantly contributes to the internal environment through which cargo is retained and released. At the nanoscale, however, mucin additionally becomes part of the carrier–biological interface, allowing its charge, glycosylation, molecular recognition sites, and amphiphilic characteristics to influence colloidal and cellular interactions directly.

Different engineering strategies have been used to produce mucin‐based nanoparticles. The DNA‐programmed carriers developed by Kimna et al. demonstrate one approach in which nanoscale mucin architecture is combined with sequence‐specific molecular control [11]. A complementary strategy was reported by Lutz et al., who developed a pH‐stable mucin nanoparticle system capable of accommodating both hydrophobic and hydrophilic compounds [40]. In that system, mucin was chemically functionalized and the resulting particles were photocrosslinked, demonstrating that covalent stabilization principles initially applied to bulk networks could also be translated to nanoscale architectures.

Miniaturization therefore changes the parameters governing delivery performance. While bulk systems are strongly influenced by macroscopic swelling and matrix diffusion, nanoparticle systems additionally depend on particle size, colloidal stability, surface chemistry, glycan presentation, cargo localization, cellular interaction, and degradation [11, 40].

An important terminology distinction should be maintained. Mucin‐based nanoparticles are not equivalent to conventional nanoparticles that merely interact with mucus or purified mucin. The extensive literature on mucus‐penetrating or mucoadhesive nanoparticles commonly uses mucin as a biological barrier or interaction substrate. Such systems fall outside the strict definition used in this review unless mucin itself constitutes a structural or functional component of the carrier [47].

Within mucin‐based nanoparticles, substantial diversity also exists. Mucin can be chemically modified before particle formation, covalently stabilized after assembly, integrated with DNA or complementary polymers, or assembled in ways intended to preserve its native glycosylated character [4, 11, 40]. This latter concept led to the emergence of mucosomes as a particularly distinctive subclass of mucin‐based nanocarriers.

### 3.6. Mucosomes: Intrinsically Functional Glycosylated Nanocarriers

The introduction of mucosomes by Butnarasu et al. in 2022 represented a distinct development in the evolution of mucin‐based nanoparticles [4]. Mucosomes were introduced as intrinsically mucoadhesive, glycosylated mucin nanoparticles designed as a multidrug delivery platform. Unlike systems in which mucin is merely deposited as a coating onto a preexisting nanoparticle core, mucin constitutes the fundamental material from which the carrier itself is formed.

The original mucosomes were produced from PGM using a one‐pot process based on protein desolvation followed by crosslinking. The resulting particles were approximately 150–300 nm in size and retained accessible glycan structures following nanoparticle formation [4]. Importantly, the platform was capable of incorporating cargos with different physicochemical characteristics, exhibited mucoadhesive behavior, could be lyophilized, and showed favorable cytocompatibility under the investigated experimental conditions. Cellular experiments further demonstrated internalization of mucosomes and partial colocalization with lysosomal compartments.

The conceptual significance of mucosomes therefore extends beyond their nanoscale dimensions. Their design attempts to translate mucin’s intrinsic biological functionality directly into the nanoparticle interface. Surface‐accessible glycans can participate in biological interactions, while the mucin‐based composition contributes molecular‐binding and mucoadhesive characteristics [48]. Functionality is therefore partly encoded within the material from which the carrier is constructed rather than being introduced exclusively through postformulation surface modification [4].

This distinction raises an important conceptual question: are mucosomes simply another formulation of mucin nanoparticles, or do they represent a distinct class of intrinsically functional nanocarriers? Based on the evidence currently available, it is more appropriate to regard mucosomes as a specialized subclass within the broader family of mucin‐based nanoparticles, distinguished by the deliberate preservation and exploitation of mucin‐associated glycosylation and interfacial biological functionality rather than by particle size alone.

Available mucosome studies have demonstrated particle formation, cargo incorporation, glycan accessibility, mucoadhesion, cytocompatibility, cellular uptake, and selected antimicrobial functions [4, 10]. However, the evidence base remains dominated by in vitro and model‐system experiments. Key controls for future studies include matched nonmucin carriers, uncrosslinked or glycan‐modified controls, endotoxin‐controlled mucin preparations, and direct assessment of whether observed biological effects arise from mucin itself, the crosslinking chemistry, or the loaded cargo. Quantitative biodistribution, degradation, systemic exposure, repeat‐dose toxicology, and immune‐reactivity studies remain limited.

At the same time, mucosomes remain a comparatively young platform, with a substantially smaller evidence base than established synthetic and natural polymeric nanoparticle systems [4, 10]. Questions concerning batch‐to‐batch variability of the mucin starting material, reproducibility of glycan presentation, crosslinking chemistry, sterilization, long‐term storage stability, manufacturing scale‐up, biodegradation, pharmacokinetics, and in vivo fate therefore remain important areas for future investigation. Consequently, the conceptual attractiveness of intrinsically functional mucin nanocarriers should be distinguished from their current level of translational maturity.

### 3.7. From Structural Engineering to Programmable Delivery: An Evolutionary Perspective

Collectively, the development of mucin‐based delivery systems reveals a progressive evolution across material architecture, engineering sophistication, and pharmaceutical functionality. At the architectural level, mucin has progressed from a component of bulk drug‐retaining matrices and three‐dimensional hydrogels toward hybrid materials, microparticles, and nanoscale carriers [4, 7, 40]. Duffy et al., for example, demonstrated the feasibility of transforming mucin into covalently crosslinked hydrogels capable of sustained delivery of chemically distinct therapeutic compounds, whereas subsequent studies translated mucin into increasingly sophisticated nanoscale architectures [4, 7, 40].

From an engineering perspective, the field has advanced from native or chemically stabilized mucin toward hybrid, dynamically crosslinked, and molecularly programmed materials. Covalent crosslinking initially provided a means of improving structural persistence and controlling network properties [7], while hybrid systems exploited complementary polymers and reversible interactions to expand material functionality [42]. More recently, dynamic glycan‐mediated interactions and DNA‐mediated crosslinking have enabled reversible, self‐healing, or molecularly triggered material behavior [9, 43].

At the functional level, these developments have expanded the role of mucin from passive cargo retention and sustained release toward controlled and stimulus‐responsive delivery, molecular recognition, and intrinsically functional carrier systems [4, 7, 11, 40]. Particularly important is the transition from systems that simply regulate drug diffusion to materials capable of converting environmental or molecular information into a delivery response. DNA‐programmed mucin systems exemplify this concept by coupling sequence‐specific molecular recognition to cargo release or material disassembly [9, 11], while mucosomes extend functional engineering to the nanoparticle interface through preservation and exploitation of mucin‐associated glycosylation [4].

A useful way to interpret this progression is therefore through three parallel evolutionary axes. Architecturally, mucin‐based delivery systems have progressed from bulk matrices toward particulate and nanoscale carriers [4, 7, 40]. From an engineering perspective, the field has advanced from native mucin toward stabilized, hybrid, dynamically crosslinked, and molecularly programmed materials [7, 9, 42, 43]. Functionally, these developments have expanded the role of mucin from passive drug retention and sustained release toward controlled and stimulus‐responsive delivery, molecular recognition, and intrinsically functional carrier systems [4, 7, 40]. Together, these parallel developments demonstrate that increasing sophistication in mucin‐based drug delivery reflects not simply miniaturization, but the progressive integration of material architecture, molecular engineering, and biological functionality.

Importantly, carrier architecture and mucin chemistry cannot be considered independently. Within bulk matrices and hydrogels, mucin‐associated interactions contribute to hydration, network organization, cargo retention, and diffusion, whereas their translation to nanoscale carriers introduces additional considerations related to particle stability, surface chemistry, glycan presentation, and carrier–biological interactions [1, 2, 40]. Similarly, mucin‐associated glycans can participate directly in material organization and reversible interactions within engineered networks [42, 43], whereas their presentation at the surface of mucin nanoparticles can become an intrinsic component of the carrier–biological interface [4].

This relationship also highlights why increasing structural complexity should not automatically be equated with superior pharmaceutical performance. A nanoscale carrier is not inherently preferable to a hydrogel, nor is a molecularly programmed material necessarily advantageous over a simpler matrix. Rather, the optimal architecture depends on the intended therapeutic function. Bulk matrices and hydrogels may be particularly suitable for localized and sustained delivery; hybrid materials can provide additional mechanical or environmental control; molecularly programmed systems offer the possibility of highly specific responsiveness; and nanoparticles provide access to nanoscale transport and greater involvement of mucin‐associated surface properties in biological interactions [9, 11, 40].

Accordingly, the evolution of mucin‐based delivery platforms should not be viewed simply as a transition from older to newer carriers, but as an expansion of the pharmaceutical design space available for exploiting mucin. The critical question is therefore no longer simply whether mucin can be transformed into a drug delivery material, but how its molecular characteristics can be deliberately integrated with carrier architecture to achieve a defined pharmaceutical function. This requires understanding how mucin interacts with therapeutic cargo, how those interactions are altered by engineering and formulation, and how they ultimately influence drug loading, retention, and release.

The following section therefore examines drug–mucin interactions and their consequences for cargo loading, retention, and controlled release, providing the mechanistic link between mucin engineering and therapeutic performance.

## 4. Smart Mucin‐Based Drug Delivery: What Makes a Mucin Carrier “Smart”?

For consistency throughout this review, passive/sustained delivery refers to release governed by diffusion, swelling, degradation, or matrix relaxation without a defined trigger; environmentally responsive delivery denotes a change in carrier behavior caused by local physicochemical conditions such as pH, ionic strength, or enzymatic activity; externally triggered delivery requires an applied stimulus such as light, heat, ultrasound, or another externally controlled input; molecularly programmed delivery incorporates a recognition element that converts a defined molecular signal into a predetermined material or therapeutic response; and on‐demand delivery is reserved for systems in which a discrete trigger initiates release or carrier transformation at the required time [49–51]. These categories are mechanistically distinct and are used accordingly below.

The concept of “smart” mucin‐based drug delivery extends beyond conventional sustained or controlled release. In its simplest form, an engineered mucin carrier can regulate drug retention and release through its network structure, swelling, or degradation; however, such behavior does not necessarily constitute true responsiveness. A more advanced level is reached when the carrier changes its properties in response to a defined physiological stimulus [49–51].

This principle has been demonstrated in pH‐responsive mucin‐containing hydrogels, in which changes in ionization and network swelling modulate drug loading and release under different pH conditions. Examples include quince/mucin co‐poly(methacrylate) hydrogels for acyclovir [44], chitosan/mucin hydrogel microparticles for tofacitinib [41], and *Delonix regia*/mucin co‐poly(acrylate) hydrogels for metformin [46]. These systems represent an important transition from passive release toward environment‐responsive delivery, although their response is governed mainly by relatively broad physicochemical changes rather than recognition of a disease‐specific molecular signal.

A substantially higher level of control is achieved when molecular recognition is incorporated directly into the mucin carrier. Kimna et al. introduced DNA‐functionalized mucin nanoparticles in which complementary oligonucleotide interactions served as programmable elements controlling particle stability and cargo release. Importantly, an endogenous microRNA sequence associated with cancer could act as the molecular trigger, enabling autonomous intracellular drug release in cells containing the relevant pathophysiological marker [11]. The same general principle has subsequently been extended to macroscopic mucin networks: DNA‐crosslinked mucin hydrogels can undergo strand displacement–mediated network destabilization and gel‐to‐sol transition, allowing triggered release of entrapped particles [9]. These approaches move mucin delivery beyond environmental sensitivity toward sequence‐specific recognition and on‐demand material response, as conceptually illustrated in Figure 4.

**FIGURE 4:**
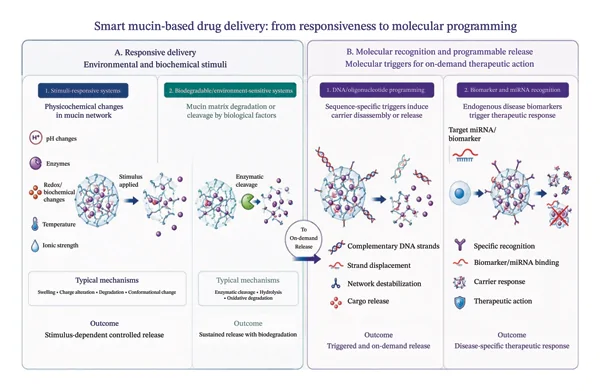
Hierarchy of smart mucin‐based drug delivery from environmental responsiveness to molecular programming. Mucin‐based systems can achieve stimulus‐responsive release through physicochemical changes or biodegradation, whereas molecularly programmed platforms use sequence‐specific or biomarker recognition to trigger carrier transformation and therapeutic release. This progression distinguishes environmentally responsive delivery from molecularly selective, on‐demand therapeutic action.

Recent developments further suggest a transition from using nucleic acids merely as external triggers toward integrating molecular recognition with therapeutic activity itself. In 2026, mucin glycoprotein nanoparticles were engineered using synthetic DNA strands that simultaneously stabilized the nanoparticles and provided sequence‐specific binding sites for oncogenic miRNA‐21. The resulting system inhibited miRNA‐21 activity and demonstrated antitumor effects in vivo, illustrating how molecular recognition can become an intrinsic component of the therapeutic mechanism rather than simply a release switch [11]. Such systems represent an important conceptual advance toward programmable mucin nanomedicine, in which carrier architecture, molecular sensing, and therapeutic function are increasingly integrated.

Nevertheless, the term “smart” should be applied cautiously. Current mucin‐based systems span a continuum from passive and controlled delivery to environmentally responsive materials, trigger‐responsive systems, and genuinely molecularly programmed carriers. Most reported formulations remain at the controlled or physicochemically responsive end of this spectrum, whereas examples capable of recognizing a specific disease‐associated molecular signal and converting that recognition into a defined therapeutic response remain comparatively limited. Even the most advanced systems require broader validation of trigger specificity, performance in complex biological environments, in vivo efficacy, and reproducibility before autonomous disease‐responsive delivery can be considered established. Thus, the current evolution of the field can be summarized as controlled release ⟶ environmental responsiveness ⟶ molecular recognition ⟶ programmable therapeutic response, with the latter representing the emerging frontier of smart mucin‐based drug delivery.

## 5. Therapeutic Applications of Mucin‐Based Drug Delivery Systems

The therapeutic scope of mucin‐based drug delivery has expanded from conventional small‐molecule encapsulation toward applications in which the biological and interfacial properties of mucin contribute directly to therapeutic performance. Current studies are particularly concentrated at mucosal surfaces, where mucin‐based carriers may combine local retention with controlled delivery and biological interactions, but emerging work also demonstrates their ability to accommodate antibiotics, poorly soluble molecules, monoclonal antibodies, and nucleic acid therapeutics. Accordingly, the most relevant applications can be grouped into mucosal and localized therapy, antimicrobial and biofilm treatment, and advanced molecular therapeutics, as summarized in Figure 5.

**FIGURE 5:**
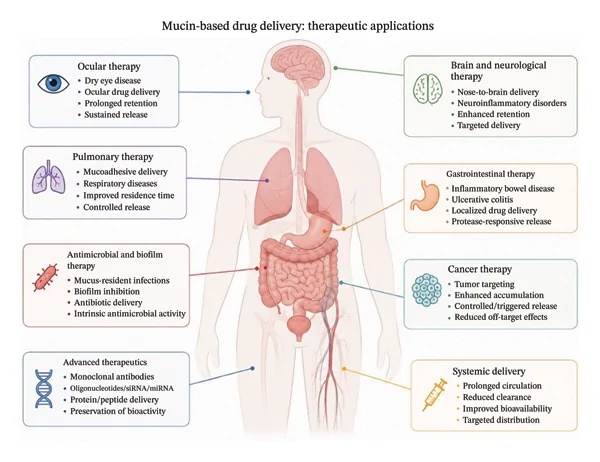
Therapeutic applications of mucin‐based drug delivery systems. Engineered mucin platforms are being investigated for gastrointestinal, ocular, pulmonary, antimicrobial/antibiofilm, cancer, and biologic/nucleic acid delivery, exploiting mucin‐associated mucoadhesion, molecular interactions, and localized or responsive release. Current evidence remains predominantly preclinical, ranging from in vitro and ex vivo studies to limited in vivo validation.

### 5.1. Mucosal and Localized Drug Delivery

Mucosal sites represent a particularly logical application for mucin‐based carriers because their material properties can be exploited within the biological environment in which mucins naturally function [52]. Recent examples illustrate this potential in both gastrointestinal and ocular delivery [53, 54]. In inflammatory bowel disease, mucin‐based hydrogels have been used for localized delivery of anti‐TNF‐α monoclonal antibodies, preserving antibody bioactivity while providing controlled release that increased under proteolytic conditions. Intrarectal administration in a murine colitis model also supported enhanced local antibody delivery relative to antibody solution [55]. In ocular delivery, alginate‐stabilized mucin nanoparticles containing coenzyme Q10 achieved high encapsulation, sustained release for up to four days, strong ocular mucoadhesion, and improvement of dry‐eye manifestations in vivo [56]. Together, these studies highlight localized retention and prolonged therapeutic exposure at mucosal interfaces as important application advantages of mucin‐based materials.

### 5.2. Antimicrobial and Biofilm‐Targeted Therapy

Antimicrobial delivery is another particularly promising application because mucin can contribute biological functionality in addition to serving as a drug carrier [57]. Ciprofloxacin‐loaded mucosomes have been evaluated against *Pseudomonas aeruginosa* and *Staphylococcus aureus*, where the intrinsic bacteriostatic properties of the mucin nanoparticles complemented antibiotic activity. Empty mucosomes inhibited bacterial growth by up to 70%, while ciprofloxacin‐loaded mucosomes reduced the ciprofloxacin minimum inhibitory concentration against *S. aureus* by up to 50%; the systems also inhibited biofilm formation, reduced established biofilm biomass, and facilitated ciprofloxacin transport in a three‐dimensional mucus model [10]. This represents an important conceptual advantage of mucin‐based delivery: the carrier can potentially cooperate with the therapeutic cargo rather than functioning as an inert vehicle, making mucus‐associated infections and biofilms particularly attractive targets.

### 5.3. Advanced Therapeutics: Biologics and Nucleic Acids

The application of mucin carriers is increasingly extending beyond conventional small molecules toward biologics and nucleic acid therapeutics. The localized delivery of functional anti‐TNF‐α monoclonal antibodies from mucin‐based hydrogels demonstrates that these materials can accommodate large protein therapeutics while preserving their biological activity [55]. At the nanoscale, mucin glycoprotein nanoparticles have been engineered using synthetic DNA strands that simultaneously stabilize the carrier and provide sequence‐specific recognition of oncogenic miRNA. The resulting antisense system inhibited miRNA‐21 and suppressed tumor growth in vivo, demonstrating the potential of mucin nanoparticles for molecularly selective nucleic acid therapy [11]. These findings broaden the application landscape from conventional drug delivery toward therapeutic proteins, antisense agents, and potentially other RNA‐based or precision therapeutics.

Table 3 summarizes representative mucin‐based delivery studies and explicitly separates formulation characteristics from the level of biological evidence. This distinction is important because much of the field remains preclinical, and promising in vitro functionality should not be interpreted as equivalent to demonstrated in vivo therapeutic benefit.

**TABLE 3:** Representative therapeutic applications of mucin‐based drug delivery systems and level of experimental evidence.

Mucin source/system	Formulation and cargo	Route/intended site	Key characteristics/release	Model	Principal reported outcome	Evidence level	References
Porcine gastric mucin	Mucosomes; multidrug platform	Mucosal/general nanocarrier	∼150–300 nm; accessible glycans; mucoadhesive; lyophilizable	Cellular and physicochemical models	Cargo loading across different physicochemical classes; cytocompatibility and uptake demonstrated	In vitro	[4]
Mucin‐based mucosomes	Ciprofloxacin‐loaded mucosomes	Mucus‐associated infection/biofilm	Antibiotic delivery with intrinsic mucin‐particle biological activity	*P. aeruginosa* and *S. aureus*; 3D mucus model	Growth and biofilm inhibition; improved ciprofloxacin performance in investigated models	In vitro/model system	[10]
Mucin + chitosan	pH‐responsive hydrogel microparticles; tofacitinib	Oral/intestinal	pH‐responsive swelling and controlled release	In vitro characterization and toxicological screening	Controlled pH‐dependent delivery demonstrated	In vitro	[41]
Mucin‐containing copolymer hydrogel	Acyclovir sodium	Oral/localized controlled delivery	pH‐dependent swelling and controlled release	In vitro	Environment‐responsive release demonstrated	In vitro	[41, 44]
Mucin‐containing copolymer hydrogel	Metformin HCl	Oral	pH‐responsive swelling; sustained release	In vitro	Controlled/sustained release demonstrated	In vitro	[46]
Porcine intestinal mucin + PEG‐thiol	Hydrogel; anti‐TNF‐α monoclonal antibody	Intrarectal/colon	Controlled antibody release; protease‐sensitive behavior	Murine colitis model plus in vitro release	Enhanced local antibody delivery with therapeutic evaluation	In vitro + in vivo	[55]
Mucin + alginate nanoparticles	Coenzyme Q10	Topical ocular	> 96% encapsulation; sustained release up to 4 days; strong mucoadhesion	Ocular in vivo evaluation	Improvement of dry‐eye manifestations reported	In vitro + in vivo	[56]
Mucin glycoprotein nanoparticles + DNA	Antisense targeting miRNA‐21	Cancer/intracellular	Sequence‐selective recognition and therapeutic oligonucleotide function	Cellular studies and tumor model	miRNA‐21 inhibition and antitumor activity reported	In vitro + in vivo	[11]

*Note:* Only the level of evidence directly demonstrated in the cited studies should be interpreted as established; broader clinical utility remains prospective.

### 5.4. Emerging Opportunities and Therapeutic Positioning

Despite this expanding application landscape, not every therapeutic cargo necessarily benefits from a mucin‐based carrier. The strongest rationale exists where mucin provides a function that conventional inert polymers do not readily reproduce, including interaction with mucus and mucosal tissues, glycan‐mediated biological interactions, intrinsic antimicrobial effects, biomolecular binding, or incorporation of programmable molecular recognition. Current evidence therefore appears particularly compelling for localized mucosal disease, infection and biofilms, ocular therapy, inflammatory disorders, and molecularly targeted therapeutics, whereas evidence supporting broader systemic applications remains comparatively limited. Future studies should consequently focus not merely on demonstrating that another drug can be loaded into mucin, but on establishing a clear therapeutic advantage attributable to the mucin component itself. This distinction will be essential for defining where mucin‐based biomaterials can progress from technically feasible carriers to therapeutically meaningful delivery platforms.

## 6. From Smart Mucin Materials to Pharmaceutical Translation: Challenges and Future Outlook

### 6.1. What Is Still Holding Mucin‐Based Delivery Back?

Despite the increasing sophistication of mucin‐based delivery systems, their translation toward pharmaceutical products remains at an early stage. A central challenge is the intrinsic heterogeneity of mucin as a biologically derived material. Mucin composition can vary with biological source and purification method, while differences in molecular structure and glycosylation may directly influence physicochemical behavior, molecular interactions, and biological functionality [1, 2]. This variability becomes particularly important for advanced carriers because the properties being exploited—including hydration, molecular binding, glycan‐mediated interactions, and responsiveness—depend on preservation of the mucin macromolecule itself. Chemical modification, crosslinking, and nanoscale processing introduce additional variables, creating challenges related to batch‐to‐batch reproducibility, structural integrity, stability, sterilization, and scale‐up. Consequently, although current studies establish the feasibility of constructing increasingly functional mucin carriers, material definition and reproducible performance remain major obstacles to their pharmaceutical translation [58].

Biological purity and immunological safety represent additional translational challenges, particularly for animal‐derived mucins. Tissue‐derived and commercial mucin preparations may retain residual cellular material, nucleic acids, associated proteins, microbial products, and bacterial endotoxins such as lipopolysaccharide (LPS), any of which can confound biological evaluation if not quantified and controlled [1, 2]. Endotoxin is especially important because LPS can activate TLR4‐dependent innate immune signaling; therefore, inflammatory responses observed with a mucin formulation cannot automatically be attributed to the mucin macromolecule itself. Source‐dependent glycosylation also raises the possibility of host recognition of xenogeneic glycan epitopes [59].

Accordingly, pharmaceutical development should incorporate validated endotoxin testing and clearance, bioburden control, assessment of residual proteins and nucleic acids, and characterization of potentially immunoreactive glycan structures. At the same time, purification must preserve the glycosylation and molecular integrity responsible for mucin functionality [1, 2].

Yan et al. [60] showed that deliberate modification of mucin glycans altered the immune activity of mucin hydrogels, underscoring that glycan composition is a functional safety attribute rather than merely a structural feature. Recombinant mucins and synthetic mucin mimics may offer improved compositional definition, contaminant control, and batch reproducibility, although they must still be validated for functional equivalence to native mucin and for their own immunological safety [59]. Future in vivo studies should therefore distinguish intrinsic mucin bioactivity from responses caused by source‐associated contaminants and establish material‐specific host‐reactivity profiles before clinical translation.

### 6.2. What Is Needed to Move From Proof of Concept to a True Drug Delivery Platform?

Future research should move beyond demonstrating the feasibility of mucin‐based hydrogels or nanoparticles and instead establish when and why mucin provides a meaningful advantage over established delivery materials. This requires standardized characterization of mucin source, subtype, purity, molecular‐weight distribution, glycosylation, and functional integrity, together with systematic structure–property–performance studies linking these attributes to carrier behavior [1, 2, 59]. Greater emphasis should also be placed on sterilization, storage stability, scalable manufacturing, biodegradation, pharmacokinetics, biodistribution, immunological safety, and long‐term biocompatibility rather than predominantly on physicochemical characterization and short‐term in vitro performance [1, 2].

For responsive and programmable systems, functionality demonstrated under simplified experimental conditions must additionally be validated for specificity, robustness, and therapeutic relevance in complex biological environments. Ultimately, direct comparison with established polymeric or lipid‐based carriers will be necessary to determine whether mucin provides a distinct therapeutic or functional advantage.

From a translational perspective, a pharmaceutical quality‐control framework for mucin‐based systems should define critical material attributes and corresponding acceptance criteria for mucin identity and source, molecular‐weight distribution, glycosylation profile, residual impurities, endotoxin and bioburden, residual crosslinkers or processing reagents, carrier characteristics, drug content, release behavior, and potency‐related functionality [1, 59, 60]. Sterilization procedures should preserve glycan presentation, molecular integrity, rheological or colloidal properties, and cargo stability, while stability studies should evaluate physicochemical degradation, aggregation, microbial quality, and functional performance throughout the intended shelf life. For systemically exposed or long‐residence formulations, pharmacokinetics, biodistribution, biodegradation, local and systemic toxicity, immunogenicity, and repeat‐dose safety should accompany efficacy evaluation. Regulatory translation will therefore depend on reproducible manufacturing, validated analytical methods, defined product specifications, stability‐indicating assays, and evidence that mucin provides sufficient functional benefit to justify the additional complexity associated with a biologically derived material.

### 6.3. Future Direction

An important route toward overcoming these limitations may be the transition from heterogeneous naturally isolated mucins toward better‐defined and molecularly engineered mucin materials [59]. Recombinant technologies already offer the possibility of producing sequence‐defined mucins, while advances in engineered cellular production, metabolic glycoengineering, and chemoenzymatic modification provide opportunities to control or redesign mucin glycosylation and thereby tune biological functionality [61]. At the carrier level, the next generation of mucosomes and related nanostructures could increasingly integrate the intrinsic biological properties of mucin with molecular sensing, biomarker recognition, and programmed therapeutic responses. Recent developments support this trajectory: mucosomes have demonstrated the ability to preserve biologically relevant mucin–bacteria interactions while delivering antibiotics [10], whereas mucin‐based hydrogels have enabled localized delivery of therapeutic monoclonal antibodies [55, 59]. The critical future advance, however, should not be greater complexity for its own sake. Progress toward smart medicine will depend on developing defined, reproducible, scalable, biologically validated, and therapeutically superior mucin systems in which added functionality translates into measurable clinical benefit.

The pathway toward translation can therefore be envisioned as

 Defined mucin ⟶ standardized engineering ⟶ reproducible carrier ⟶ validated biological functionality ⟶ disease‐specific responsiveness ⟶ in vivo efficacy and safety ⟶ scalable manufacturing ⟶ clinical translation.

## 7. Conclusion

Mucin has progressed from being viewed primarily as a component of biological barriers to becoming a versatile building block for engineered drug delivery systems. Advances in crosslinking, hybridization, nanoscale assembly, and molecular programming have progressively expanded mucin platforms from passive matrices toward responsive and increasingly programmable therapeutic systems. Nevertheless, the field remains predominantly preclinical, and its future will depend less on producing additional proof‐of‐concept formulations than on resolving material heterogeneity, reproducibility, manufacturing, safety, and comparative therapeutic performance. The convergence of recombinant mucin production, glycoengineering, nanotechnology, and molecular programming provides a particularly promising route toward carriers whose biological functionality can be deliberately designed rather than inherited unpredictably from the source material. If these advances can be coupled with rigorous in vivo validation and scalable pharmaceutical development, mucin‐based systems may progress from promising smart biomaterials toward clinically relevant smart medicine.

## Funding

This research received no specific grant from any funding agency in the public, commercial, or not‐for‐profit sectors.

## Conflicts of Interest

The author declares no conflicts of interest.

## Data Availability

No original datasets were generated in this review article. All information discussed in the manuscript is derived from previously published studies, which are appropriately cited in the reference list.
